# Amphiphilic Peptides for Efficient siRNA Delivery

**DOI:** 10.3390/polym11040703

**Published:** 2019-04-17

**Authors:** Saghar Mozaffari, Emira Bousoik, Farideh Amirrad, Robert Lamboy, Melissa Coyle, Ryley Hall, Abdulaziz Alasmari, Parvin Mahdipoor, Keykavous Parang, Hamidreza Montazeri Aliabadi

**Affiliations:** Center for Targeted Drug Delivery, Department of Biomedical and Pharmaceutical Sciences, Chapman University School of Pharmacy, Harry and Diane Rinker Health Science Campus, Irvine, CA 92618, USA; mozaf100@mail.chapman.edu (S.M.); bouso100@mail.chapman.edu (E.B.); amirr100@mail.chapman.edu (F.A.); robertlamboy@gmail.com (R.L.); coyle109@mail.chapman.edu (M.C.); hall222@mail.chapman.edu (R.H.); alasm103@mail.chapman.edu (A.A.); mahdi102@mail.chapman.edu (P.M.)

**Keywords:** siRNA, peptides, breast cancer, hydrophobic modification, cell internalization

## Abstract

A number of amphiphilic cyclic peptides—[FR]_4_, [WR]_5_, and [WK]_5_—containing hydrophobic and positively-charged amino acids were synthesized by Fmoc/tBu solid-phase peptide methods and evaluated for their efficiency in intracellular delivery of siRNA to triple-negative breast cancer cell lines, MDA-MB-231 and MDA-MB-468, in the presence and absence of 1,2-dioleoyl-sn-glycero-3-phosphoethanolamine (DOPE). Among the peptides, [WR]_5_, which contains alternate tryptophan (W) and arginine (R) residues, was found to be the most efficient in the delivery of siRNA by improving the delivery by more than 3-fold when compared to other synthesized cyclic peptides that were not efficient. The data also showed that co-formulation of [WR]_5_ with lipid DOPE significantly enhanced the efficiency of siRNA delivery by up to ~2-fold compared to peptide alone. Based on the data indicating the efficiency of [WR]_5_ in siRNA delivery, peptides containing arginine residues on the ring and tryptophan residues on the side chain, [R_6_K]W_6_ and [R_5_K]W_5_, were also evaluated, and demonstrated improved delivery of siRNA. The presence of DOPE again enhanced the siRNA delivery in most cases. [WR]_5_, [R_5_K]W_5_, and [R_6_K]W_6_ did not show any significant toxicity in MDA-MB-231, MDA-MB-468, and AU565 WT cells at N/P ratios of 20:1 or less, in the presence and absence of DOPE. Silencing of kinesin spindle protein (KSP) and Janus kinase 2 (JAK2) was evaluated in MDA-MB-231 cells in the presence of the peptides. The addition of DOPE significantly enhanced the silencing efficiency for all selected peptides. In conclusion, peptides containing tryptophan and arginine residues were found to enhance siRNA delivery and to generate silencing of targeted proteins in the presence of DOPE.

## 1. Introduction

Post-transcriptional regulation of protein expression is an important intracellular mechanism that relies on the interference of non-coding RNAs (RNAi) and a protein complex containing Argonaute protein (AGO) and cofactors [1]. Human AGO protein subfamily consists of AGO1, 2, 3, and 4 and bind to microRNAs (miRNAs) and small interfering RNAs (siRNAs) [2]. siRNA would lose one of the strands (known as passenger strand), and the guide strand is incorporated into AGO-containing protein complex (known as RNA-induced silencing complex or RISC). The guide strand directs the RISC complex to the target mRNA, and the binding of the guide strand to the mRNA (based on complementary sequences) will trigger the cleavage and therefore, degradation of mRNA, which is known as “silencing” [3]. This intracellular mechanism has been the inspiration for different exogenous small RNA interfering strategies for protein silencing. However, delivering nucleic acids into the cell is challenging due to their size, hydrophilic nature, and the negative charges created by phosphate groups.

Throughout the last two decades, efficient non-viral siRNA delivery strategies have been pursued, which include different physical approaches [4]. Physical approaches such as electro-permeabilization and microinjection have been utilized in the delivery of siRNA. The use of physical strategies can be highly effective in siRNA delivery to a small population of cells [5]; however, these approaches can jeopardize the integrity of cellular membrane and are restricted as in vitro investigational tools [6]. Therefore, molecular carriers such as peptide-, polymer-, and lipid-based strategies have been investigated for in vivo siRNA delivery.

One of the recent attempts in siRNA delivery is a multi-component approach known as stable nucleic acid-lipid particles (SNALPs). This approach was originally adapted from a DNA delivery protocol and is based on encapsulating the nucleic acid cargo into lipid-based liposomal-like particles with a less than 200 nm diameter [7]. This approach was first reported for targeting hepatitis B virus in a mouse model [8], and has since been studied in clinical trials (Phases I and II) for targeting polo-like kinase I (PLK1) in patients in with adrenocortical cancer [9]. Additionally, direct conjugation of *N*-acetylgalactosamine (GalNAc; a targeting moiety with high affinity for the asialoglycoprotein receptor in the liver) to siRNA or incorporating it into the siRNA delivery system has been recently reported [10]. Polyelectrolyte-gold nanoassemblies [11], polycation liposome-encapsulated calcium phosphate nanoparticles [12], folic acid-modified mesoporous silica nanocarriers [13], and folate-PEG-appended dendrimer/α-cyclodextrin conjugates [14,15] are among other recently reported strategies for siRNA delivery.

Cell-penetrating peptides (CPPs) have drawn significant attention in the drug delivery field. The intrinsic property of CPPs to deliver therapeutic molecules (nucleic acids, drugs, imaging agents) to cells and tissues in a nontoxic manner has indicated that they may be beneficial for enhancing the cellular delivery of drugs and diagnostic agents with limited cellular uptake [16]. The CPPs have been widely used in molecular pharmaceutics, due to their ability to increase cellular internalization and to alter the pharmacokinetic pattern of the drug [17]. The modification of CPPs is required to act as a delivery vector for siRNA to minimize the degradation and mediate the efficient cellular uptake.

We have previously reported several cyclic peptides with cell penetrating properties [18,19,20,21]. The linear peptides are usually more susceptible to hydrolysis by peptidases when compared with the cyclic peptides. Furthermore, the cellular uptake of many linear cell-penetrating peptides occurs predominantly through an endocytic pathway. On the other hand, cyclic peptides containing alternate tryptophan and arginine residues [WR]_5_ and [WR]_4_, and the corresponding peptide-capped gold nanoparticles were found to act as efficient molecular transporters of siRNA in human cervix adenocarcinoma (HeLa) cells. The mechanism of uptake for [WR]_4_ and [WR]_5_ was found to be endocytosis independent, since no significant difference in cellular uptake was observed in the presence of different endocytic inhibitors [18]. Flow cytometry studies showed that [WR]_5_ and [WR]_5_-capped gold nanoparticles improved the intracellular uptake of siRNA by about 2- and 3.8-fold versus siRNA alone. We also found out that both delivery platforms were less toxic when compared to lipofectamine. Fluorescence microscopy data confirmed the localization of fluorescence-labeled siRNA in cytoplasm in the presence of [WR]_5_ and [WR]_5_-capped gold nanoparticles [22]. Thus, we hypothesized that the positively charged residues on the peptide can have electrostatic interactions with negatively charged phosphate residues in the phospholipid bilayer and siRNA. Furthermore, the hydrophobic tryptophan groups could interact with hydrophobic residues in the lipid membrane.

Herein, we investigated [WR]_5_ in comparison with other cyclic peptides containing alternate hydrophobic and positively-charged residues, [FR]_4_ and [WK]_5_. The rationale of this design is based on the preliminary data that cyclic peptide [WR]_5_ containing alternative tryptophan (W) and arginine (R) residues significantly enhanced the cellular of negatively charged phosphopeptides [20] and siRNA [22]. Furthermore, we compared the siRNA delivery with cyclic peptides containing arginine residues attached with a hydrophobic chain of tryptophan [R_5_K]W_5_, and [R_6_K]W_6_. The peptides were used alone or in different ratios with siRNA for comparative studies in two triple-negative breast cancer cell lines (MDA-MB-231 and MDA-MB-468) for cyclic peptides and an additional breast cancer cell line (AU565) for [R_5_K]W_5_ and [R_6_K]W_6_.

Furthermore, we have evaluated the effect of addition of 1,2-dioleoyl-sn-glycero-3-phosphoethanolamine (DOPE) to the delivery system. Enhancing the level of interaction with lipophilic moieties of cell membrane by increasing the hydrophobic characteristics of the carrier has been a well-studied strategy in delivering nucleic acid to cytoplasm. We have previously reported using DOPE as an additional hydrophobic component for hybrid peptides. In 2017, we reported siRNA delivery to breast cancer cell lines using arginine and lysine-containing linear and cyclic peptides conjugated to a variety of fatty acids to enhance the hydrophobicity of the carrier. Our experiments showed a significant improvement in cell internalization and silencing efficiency of the siRNA after addition of DOPE to the delivery system [23]. Finally, silencing efficiency of the selected peptides was investigated for kinesin spindle protein (KSP), and Janus kinase 2 (JAK2) proteins in MDA-MB-231 cells.

## 2. Materials and Methods

### 2.1. Materials

All amino acids and resins were purchased from AAPPTec (Louisville, KY, USA) for in-house peptide synthesis. All the other chemicals and reagents used in synthesis and purification of peptides, as well as TRIzol reagent and formaldehyde (37%) were purchased from Sigma-Aldrich (St. Louis, MO, USA). 1,2-Dioleoyl-sn-glycero-3-phosphoethanolamine (DOPE) was obtained from Avanti Polar Lipids Inc. (Alabaster, AL, USA). The chemical structures of the final products were confirmed by high-resolution matrix-assisted laser desorption/ionization (MALDI) time-of-flight (MALDI-TOF, GT 0264) from Bruker Inc. (Billerica, MA, USA). Mass spectra were collected using a freshly prepared α-cyano-4-hydroxycinnamic acid (5 mg/mL) in water (0.1% TFA) and acetonitrile (0.1% TFA) in a 1:1 ration (*v*/*v*) as the matrix that was mixed with the peptides before the measurement. Hanks’ Balanced Salt Solution (HBSS), fetal bovine serum (FBS), penicillin (10,000 U/mL), and streptomycin (10 mg/mL), and SYBR Green II were provided by Life Technologies (Grand Island, NY, USA). iScript^TM^ Reverse transcription Supermix and iTaq Universal SYBR Green Supermix used in qPCR, as well as all western blot accessories (including 10% Mini-PROTEAN^®^ TGX Stain-Free™ Protein Gels, Clarity™ Western ECL Substrate, Trans-blot^®^ Turbo^TM^ Cassettes, and Trans-Blot^®^ Turbo™ Mini PVDF Transfer Packs) were obtained from Bio-Rad (Hercules, CA, USA). 4′,6-Diamidino-2-phenylindole (DAPI) was provided by Vector Laboratories (Burlingame, CA, USA). Phosphate-buffered saline (PBS, ×20 concentration), Phalloidin, Sulforhodamine 101 (Texas Red), and other reagents were purchased from VWR (Radnor, PA, USA). Scrambled negative control siRNA (Catalogue no. AM4635), 5′-carboxyfluorescein (FAM)-labeled negative control siRNA (Catalogue no. AM4620), and the siRNA targeting kinesin spindle protein (KSP; Catalogue no. AM16704) were purchased from Ambion (Austin, TX, USA). The siRNAs targeting Janus kinase 2 (JAK2) (Catalogue no. SI02659657; Sense: 5′-CCA UCA UAC GAG AUC UUA ATT-3′; Antisense: 5′-UUA AGA UCU CGU AUG AUG GCT-3′), mLST8 (Catalogue no. SI00425474; Sense: 5′-GCU ACG ACG GCG UCA ACA ATT-3′; Antisense: 5′-UUG UUG ACG CCG UCG UAG CTG-3′, and mammalian Target of Rapamycin (mTOR; Catalogue no. SI00070462; Sense: 5′-GGA GUG UUA GAA UAU GCC ATT-3′; Antisense: 5′-UGG CAU AUU CUA ACA CUC CGG 3′) were purchased from Qiagen (Valencia, CA). Primers for KSP (Forward: 5′-TCA CAA AAG CAA TGT GGA AAC CTA-3′; Reverse: 5′-TCT GTC CAA AGA TTCA TTA ACT TGC A-3′), and JAK2 (Forward: 5′-AAC TGC AGA TGC ACA TCA TTA CCT-3′; Reverse: 5′-TCG AAA TTG GGC CAT GAC A-3′) were designed and ordered from Integrated DNA Technologies (IDT; Coralville, IA, USA). Monoclonal antibody against JAK2 (Rabbit Mab 3230; catalog number: 3230) and anti-rabbit polyclonal secondary antibody HRP-linked (7074V) were supplied by Cell Signaling Technology, Inc. (Danvers, MA, USA).

### 2.2. Cell Lines

Three different breast cancer cell lines were included in this study to represent a variety of phenotypes: MDA-MB-231 (triple-negative, but expected to express EGFR; ATCC^®^ HTB-26™), MDA-MB-468 (triple-negative, but over-expresses EGFR; ATCC^®^ HTB-132™), and AU565 (over-expresses HER2, and expected to express EGFR; ATCC^®^ CRL-2351™). All cell lines were incubated for the duration of experiments in 37 °C and 5% CO_2_ level. Dulbecco’s modified Eagle’s medium low glucose (DMEM), containing L-glutamine, sodium pyruvate, and glucose was used for MDA-MB-231 and MDA-MB-468 cells. RPMI 1640 medium was used for AU565. All media were supplemented with 10% (*v*/*v*) fetal bovine serum, 100 U/mL penicillin and 100 μg/mL streptomycin. Cells were sub-cultured after reaching > 80% confluency and were discarded after ~40 passages.

### 2.3. Synthesis and Purification

Synthesis of [FR]_4_, [WR]_5_ [18], and [WK]_5_ [19] have been previously reported by us through solid-phase synthesis. Synthesis of [R_5_K]W_5_ and [R_6_K]W_6_ was performed by using Fmoc-Arg(Pbf)-OH, Fmoc-Trp(Boc)-OH, Fmoc-Lys(Boc)-OH, and Fmoc-Lys(Dde)-OH as building block amino acids. The appropriate resin, H-Arg (Pbf)-2-chlorotrityl resin (0.44 meq/g, 905 mg) was swelled in DMF under dry nitrogen for 15 min, three times. The solvent was filtered off. The next Fmoc protected amino acid (3 equiv.) was coupled to the *N*-terminal of the arginine in the presence of 2-(1H-benzotriazol-1-yl)-1,1,3,3-tetramethyluronium hexafluorophosphate (HBTU) (3 equiv.), and *N*,*N*-diisopropylethylamine (DIPEA) (6 equiv.) in DMF by agitating under dry nitrogen for 1.5 h. The reaction solution was filtered off after the completion of the coupling. The resin was washed with *N*,*N*-dimethylformamide (DMF, 15 mL, 2 × 5 min.). Fmoc deprotection was performed by using piperidine in DMF (20% *v*/*v*, 10 mL, 2 × 15 min.). The reaction solution was filtered off, and the resin was washed with DMF (15 mL, 2 × 5 min.). The subsequent amino acids were coupled and deprotected in a similar manner. Fmoc deprotection was performed on the last amino acid. Capping reaction by acetic anhydride (3 equiv.) and (DIPEA) (6 equiv.) was performed on the last amino acid. The resin was washed with DMF (15 mL, 2 × 5 min.) and methanol (15 mL, 5 min.). The resin was dried under vacuum for 4 h. The resin cleavage cocktail, dichloromethane:trifluoroethanol:acetic acid (DCM:TFE:AcOH; 35 mL:10 mL:5 mL) was freshly prepared. The cleavage cocktail was added to the resin, and the solution was mixed for 3 h. The filtrate was evaporated under low pressure. Hexane (2 × 20 mL) and DCM (2 × 15 mL) were added to the residue to remove the acetic acid from the mixture. The crude material was solidified as a white solid. The crude peptide was dried under vacuum overnight.

After the formation, the structure of protected linear peptide was confirmed by MALDI mass spectroscopy, the crude protected peptide was used for the cyclization reaction. The cyclization occurred between the free COOH side in arginine and free NH_2_ side in lysine. Anhydrous DMF (100 mL), anhydrous DCM (50 mL), *N*,*N′*-diisopropylcarbodiimide (DIC, 0.3 mmol, 188 μL), and 1-hydroxy-7-azabenzotriazole (HOAt, 0.3 mmol, 122.5 mg) were added to the crude protected linear peptide for cyclization. The solution was stirred under dry nitrogen overnight. After the formation of the cyclic peptide was confirmed by MALDI analysis, the solvents were removed under reduced pressure. The crude peptide was dried overnight. The cleavage cocktail (TFA, anisole, thioanisole, 9:1:2 *v*/*v*/*v* and 50 mg of dithiothreitol (DTT), 20 mL total volume) was added to the crude product. The mixture was stirred at room temperature for 5 h. The crude peptide was precipitated in cold diethyl ether and centrifuged. Final compounds were purified by a reversed-phase HPLC LC-20AP pump (Shimadzu Corp., Kyoto, Japan) using a gradient system of acetonitrile and water and a reversed-phase preparative column (XBridge BEH130 Prep C18; 19 × 250 mm, 10 µm particle size, Waters Corp., Milford, MA, USA). The structure of the compound was confirmed by high-resolution MALDI-TOF mass spectroscopy (GT 0264; Bruker Inc., Billerica, MA, USA).

[R_5_K]W_5_: MALDI-TOF (m/z): C_93_H_124_N_32_O_12_, calculated: 1881.0076, found: 1883.8647 [M+2H]^+^;
[R_6_K]W_6_: MALDI-TOF (m/z): C_110_H_146_N_38_O_14_, calculated: 2223.1881, found: 2225.0676 [M+2H]^+^

### 2.4. Complex Formation and Cytotoxicity of Peptide/siRNA Complexes

A Cell Counting 8 (CCK8) KIT (Biotool; Houston, TX, USA; also known as WST-8) was used to evaluate the cytotoxicity of the peptides in breast cancer cell lines (MDA-MB-231, MDA-MB-468, and AU565). Peptide/scrambled siRNA complexes were prepared by mixing siRNA and peptide in normal saline, and formed spontaneously based on inter-ionic interaction. The mixture was incubated for 30 min at room temperature to ensure complete complex formation. The ratio of peptide:siRNA was calculated based on nitrogen/phosphate (N/P) ratio. The N/P ratios were calculated using Equation (1)

(1) $$N/P=\frac{\#\;of\;Moles\;of\;peptide\times \#\;of\;protonable\;nitrogens\;in\;each\;molecule\;of\;peptide}{\#\;of\;Moles\;of\;siRNA\times \#\;of\;phosphate\;groups\;in\;each\;siRNA\;molecule}$$

The peptide/siRNA complexes were formed with a wide range of N/P ratios (from 1 to 80) and added to the cells in triplicate for a final siRNA concentration of 54 nM for all treatment groups. Lipofectamine ^®^ 2000 was used as control following manufacturer’s instructions to deliver a similar siRNA final concentration. Cells were incubated at 37 °C, 5% CO_2_, for 24 h, after which 10 μL of CCK8 reagent was added to each well, and plates were incubated at 37 °C, 5% CO_2_, for 2 h. The absorbance of each well was measured at 450 nm using the SpectraMAX M5 microplate reader. The results were normalized to cells treated with normal saline (considered as 100%) after subtracting the signal from blank wells (medium without cells in the plate with CCK-8 solution added).

### 2.5. Binding Affinity

Peptides were studied for their affinity for interionic interaction and binding to siRNA using a SYBR green dye exclusion assay that we have previously reported [23,24]. Scrambled siRNA and peptide solutions were mixed in normal saline with different N/P ratios (ranging between 0.05 to 40). Free siRNA was used as a negative control. After 30 min incubation in room temperature 200 μL of SYBR Green II dye (dilution of 1:10,000 was prepared using distilled water) was added to the samples, and the fluorescence signal was measured in 96-well black plates (λ excitation: 485 nm, λ emission: 527 nm). The percentage of peptide-bound siRNA was calculated based on Equation (2) and plotted against N/P ratio to calculate the ratio resulting in 50% binding (BR50) based on the linear portion of the graph

(2) $$\%\;siRNA\;bound\;to\;peptide=100-\left(\frac{Fluorescence\;signal\;for\;complex}{Fluorescence\;signal\;for\;free\;siRNA}\times 100\right).$$

### 2.6. Size and ξ-potential of Peptide/siRNA Complexes

In order to further characterize the peptide/siRNA particles, the hydrodynamic diameter and the surface charge of the particles were determined using a Malvern Nano ZS Zetasizer (Westborough, MA, USA) at 25 °C in disposable cuvettes and folded capillary cells, respectively. The ξ-potential of the complexes was determined at 40 V, using Smoluchowski approximation.

### 2.7. Cellular Internalization (Flow Cytometry)

The capability of the peptides in internalizing siRNA into human cells was evaluated by monitoring the uptake of fluorescence (FAM)-labeled scrambled siRNA and flow cytometry (BD-FACSVerse; BD Biosciences; San Jose, CA, USA). All three breast cancer cell lines were used for these studies and were seeded in 24-well plates (~200,000 cells per well). Peptide/siRNA complexes were prepared in N/P ratios of 20:1, 40:1, and 80:1, with a final concentration of 36 nM for the fluorescent-labeled scrambled siRNA. Cells exposed to siRNA complexes were incubated in 37 °C and standard growth conditions for 24 h, after which cells were washed with clear HBSS (×2), trypsinized, and fixed using 3.7% formaldehyde solution. Suspended cells were analyzed using FITC channel to quantify cell-associated fluorescence. In order to evaluate the potential effect of the addition of DOPE to the carrier, DOPE was added to the complexes in a peptide/DOPE molar ratio of 3:2. After each flow cytometry analysis, the percentage of cells with fluorescence signal and the mean fluorescence of the cell population was calculated based on the calibration of the signal gated with non-treated cells (as the negative control), so that the auto-fluorescence would be ~1% of the population.

### 2.8. Confocal Microscopy

Sterile cover slips were placed in the wells of 6-well plates and were treated with 2 μL of media for 2 h. The cells were then seeded on the cover slips, and after 24 h growth period, were exposed to FAM-labeled siRNA with a similar method as explained for flow cytometry experiments (N/P ratio of 20 was used for this set of experiments to avoid cytotoxicity). After 24 h, the growth media was removed, and cells were washed with clear HBSS (×3). The cells were then fixed with 3.7% formaldehyde solution in HBSS for 10 min, and were exposed to Texas Red (60 min in room temperature) and DAPI (overnight, away from light and at room temperature) to stain cell membrane and nuclei, respectively. Cover slips were examined using a Nikon A1R high definition resonant scanning confocal microscope and a NIS-Elements software (AR 4.30.02, 64bit).

### 2.9. mRNA Interference (Real-Time Polymerase Chain Reaction; RT-PCR)

Cells were exposed to peptide/siRNA complexes formed with different N/P ratios (with final siRNA concentration of 50 nM), and RNA was extracted from cells using TRIzol reagent 48 h after exposure, following the manufacturer’s instructions. One mL of TRIzol reagent was added for each 1 × 10^6^ cells, and the cell lysates were incubated at room temperature for 5 min The lysate was then treated with chloroform at *v*/*v* chloroform:TRIzol ratio of 1:5. The contents of the tubes were mixed and incubated for 2–3 min at room temperature, and the aqueous phase was collected. The total RNA was precipitated using isopropanol and was pelleted by centrifugation (12,000 g for 10 min in 4 °C). The pellet was washed with 75% ethanol, and the extracted RNA was dissolved in RNase-free water. The total extracted RNA in each sample was quantified by BioSpec-Nano (Shimadzu, Columbia, MD, USA). The cDNA was synthesized by reverse transcription of 0.5–1 μg RNA using iScript^TM^ reverse transcription Supermix and the C1000 Touch^®^ thermocycler (Bio-Rad, Hercules, CA, USA), following the manufacturer’s guidelines. A CFX96TM optical module (Bio-Rad) was used for RT-PCR analysis, and human β-actin was used as the endogenous gene to normalize the mRNA level of targeted proteins. RT-PCR was performed using iTaq universal SYBR green Supermix kit. The RT-PCR ran for 40 cycles (95 °C for 2 min., followed by 40 cycles of denaturation 95 °C for 5 s and annealing temperature 55 °C for 30 s, and a final extension step at 65 °C for 5 s). The analysis was performed by calculating ΔCT (the difference in CTs for the target mRNA and the endogenous control), ΔΔCT (based on ΔCTs observed for ‘no treatment’ and the treatment groups) and relative quantity (RQ).

### 2.10. Protein Quantification (Western Blot)

The expression of the targeted protein was analyzed by western blot. Cell lysates were prepared according to the standard protocol using RIPA buffer. Briefly, cells exposed to siRNA complexes were collected by trypsinization after 48 h of exposure and were centrifuged at 600–800 RPM for 5 min. The supernatant was discarded, and the cell pellet was washed three times with ice cold PBS. The pellet was resuspended in RIPA buffer (100 μL of buffer for 25 μL of cell pellet), and the cell lysates were then incubated on ice for one hour, during which the tubes were sonicated for 3 min every 10 min The tubes were then centrifuged for 15 min at 12,000× *g* (at 4 °C). Microtubes were pre-cooled to transfer the supernatant, and total protein concentration was determined using BSA assay. Briefly, 200 μL of work reagent (50:1 A:B) was added to 25 μL of standard and unknown samples in triplicate into a 96-well plate, and the plate was mixed on a plate shaker for 30 s. The plate was then incubated at 37 °C and 5% CO_2_ for 30 min. The absorbance was measured at 562 nm using SpectraMAX M5 microplate reader. Protein (25 μg) was loaded per well in a 10% Mini-PROTEAN^®^ TGX Stain-Free™ Protein gel using electrophoresis buffer (0.192 M glycine, 25 mM Tris, 0.1% SDS), and the electrophoresis was run for 30 min with 200 V. The gel was then transferred onto a Trans-Blot^®^ Turbo™ Mini PVDF membrane (Catalog no. 1704156). Membranes were blocked in BSA 5% for 3 h, and then incubated overnight (at 4 °C) with the primary anti-body (1:1000 in TBS-T). The membrane was then washed with TBS-T six times (5 min each time), and was subsequently incubated with the secondary HRP-linked antibody (1:1000 in TBS-T) for 1 h, followed by the washing steps. Detection was done by ECL Detect Kit using ChemiDoc imager (Bio-Rad).

## 3. Results and Discussion

### 3.1. Chemistry

**Scheme 1:**
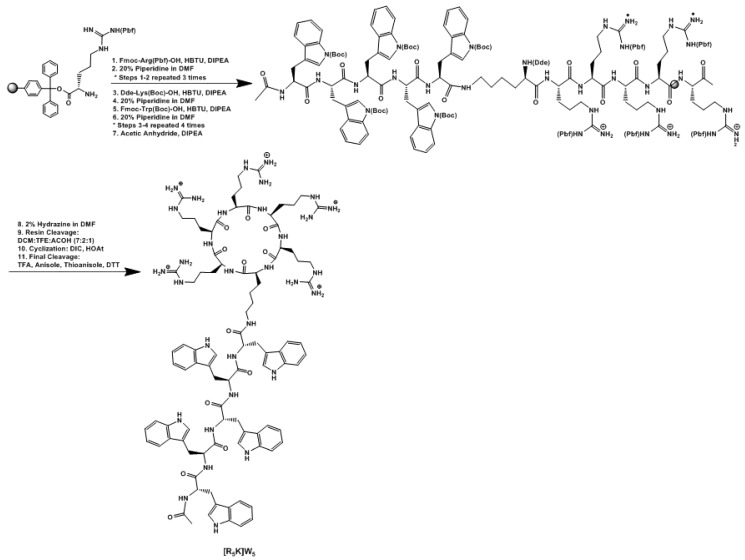
Synthesis of [R_5_K]W_5_.

As a representative example, the synthesis of [R_5_K]W_5_ is shown in Scheme 1. Synthesis of [R_5_K]W_5_ was performed by using Fmoc solid-phase peptide synthesis. The appropriate resin, H-Arg(Pbf)-2-chlorotrityl resin was used as the solid support. Fmoc-Trp(Boc)-OH, Fmoc-Arg(Pbf)-OH, and Dde-Lys(Boc)-OH were used as building block amino acids in the assembly of the peptide on the resin. After the protected linear peptide formation was confirmed by MALDI mass spectroscopy (see Supporting Information), the crude peptide was used for the cyclization reaction in the presence of anhydrous DMF, anhydrous DIC, and HOAt. The crude peptide was precipitated in cold diethyl ether and was purified by HPLC.

**Figure 1:**
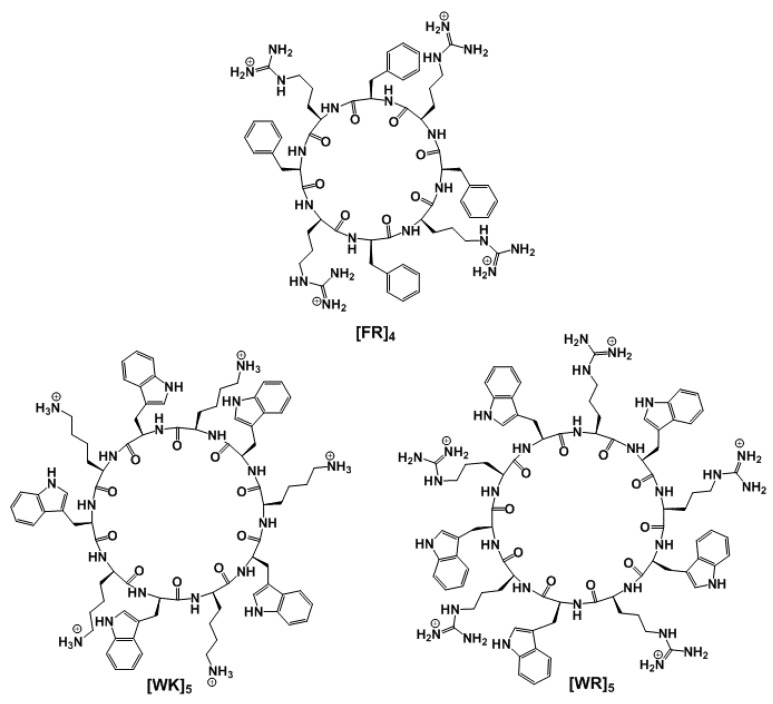
Cyclic peptides containing hydrophobic and positively-charged residues.

**Figure 2:**
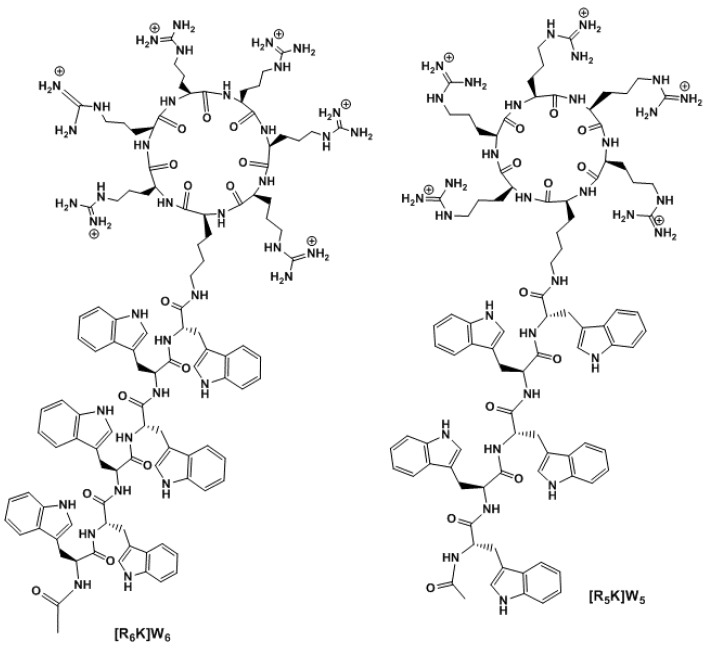
Peptides containing positively-charged residues on the ring attached to dydrophobic tryptophan chain.

The selected peptides in this study contained arginine or lysine positively-charged residues and hydrophobic tryptophan or phenylalanine. [FR]_4_, [WR]_5_, and [WK]_5_ (Figure 1) were designed and synthesized for siRNA delivery to be used alone, or in combination with DOPE. It was expected that positively-charged and hydrophobic residues in the structure can greatly improve the interaction with the negatively charged phosphate and hydrophobic chains in the cellular phospholipid bilayer, respectively, and enhance the cellular delivery of siRNA. Alternatively, we synthesized cyclic peptides containing positively-charged arginine residues attached to hydrophobic tryptophan chain, [R_5_K]W_5_ and [R_6_K]W_6_ (Figure 2). The goal was to determine whether changing the orientation of arginine and tryptophan residues and the addition of a linear hydrophobic chain can affect the siRNA delivery.

### 3.2. Cellular Internalization

**Figure 3:**
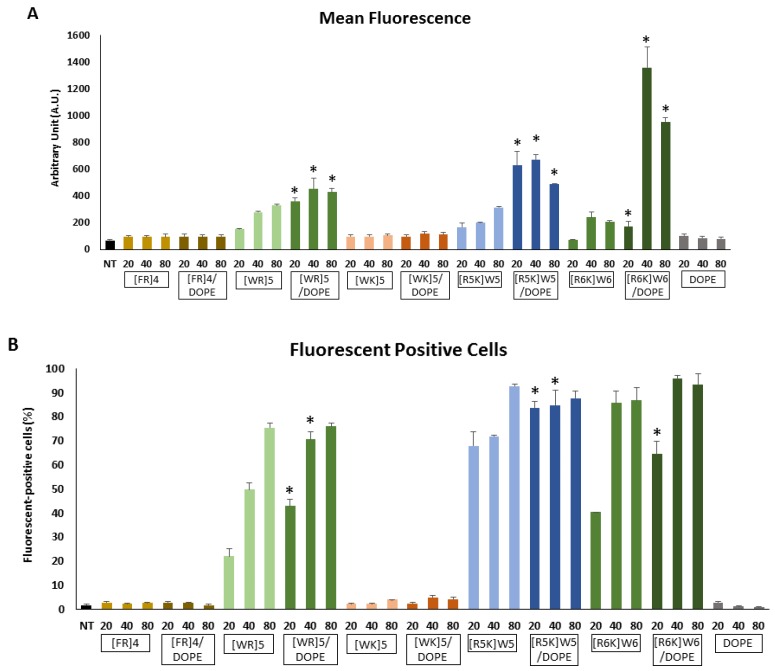

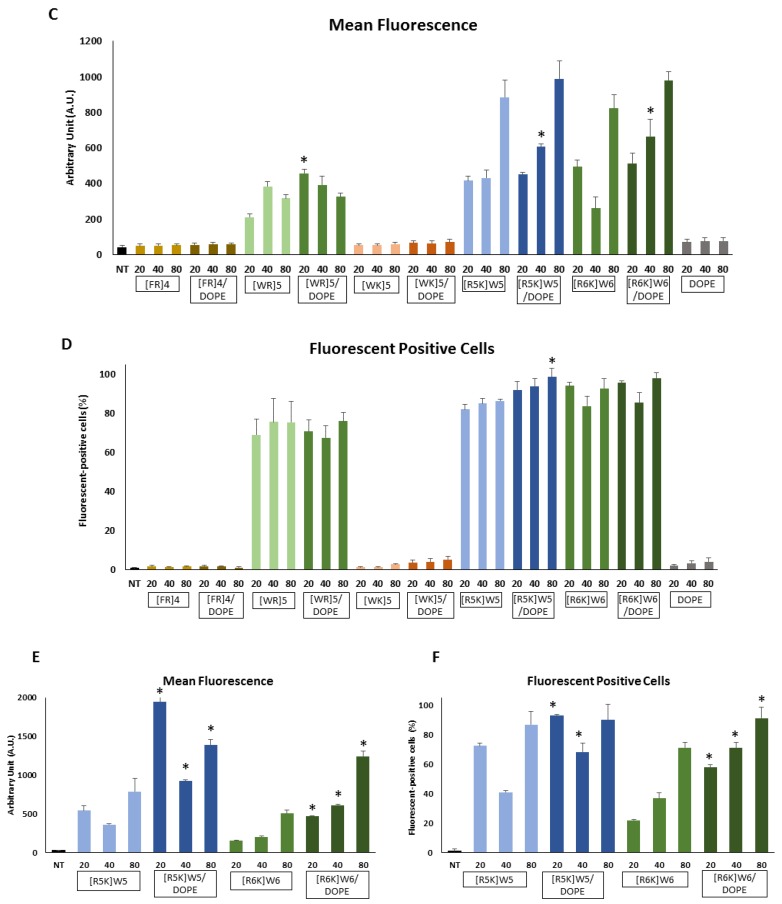
The efficacy of siRNA cell internalization with designed pepties (with and without addition of DOPE) in MDA-MB-231 Cells (**A**,**B**), MDA-MB-468 (**C**,**D**), and AU565 cells (**E**,**F**; only for [R_5_K]W_5_ and [R_6_K]W_6_ with different N/P ratios (20:1, 40:1, and 80:1) (Flow cytometry; *n* = 3). The uptake is presented as both mean fluorescence in the whole cell population (**A**,**C**,**E**) and percentage of cells identified as fluorescent positive (**B**,**D**,**F**). The “DOPE” group represents internalization of siRNA with DOPE alone. The error bars indicate standard deviation. Asterisks indicate significant difference (*p* < 0.05) with the ‘without DOPE’ corresponding group.

The study of siRNA intracellular delivery using cyclic and hybrid (positively charged ring and hydrophobic chain) peptides was performed using flow cytometry in two triple-negative breast cancer cell lines (MDA-MB-231 and MDA-MB-468) for cyclic peptides and an additional breast cancer cell line (AU565, which over-expresses HER2, and is expected to express EGFR as well) for hybrid peptides. We investigated the efficiency of three N/P ratios for each peptide, and also determined the effect of an additional hydrophobic moiety to the carrier by adding DOPE to the formulation of each peptide/siRNA complex (Figure 3). Among cyclic peptides, [FR]_4_ and [WK]_5_ showed no significant difference with cells exposed to normal saline at any studied N/P ratio. However, [WR]_5_ indicated significant efficacy in cellular internalization of siRNA. In MDA-MB-231 cells, both mean fluorescence of the whole cell population and the percentage of the cells with fluorescent signal were improved by increasing the N/P ratio. Also, the addition of DOPE significantly increased internalization of the siRNA in all three N/P ratios (Figure 3A,B). The average mean fluorescence for N/P ratio of 40 after addition of DOPE was slightly higher than N/P ratio of 80; however, the difference was not statistically significant. Generally, a similar uptake efficiency was observed for [WR]_5_ in MDA-MB-468 cells (maximum mean fluorescence of 459 was achieved with [WR]_5_/DOPE at N/P ratio of 20, compared to maximum mean fluorescence of 454 for [WR]_5_/DOPE at N/P ratio of 40 in MDA-MB-231 cells). However, while similar improved internalization was observed with the addition of DOPE to the delivery system, the trend among the different N/P ratios was different. Without DOPE, an N/P ratio of 40 showed the highest mean fluorescence, where after the addition of DOPE, an N/P ratio of 20 showed the highest efficiency in terms of mean fluorescence. There was no significant difference in the percentage of cells positive for fluorescence signal in MDA-MB-468 cells among [WR]_5_ study groups, which was partially due to overall high percentages (69–77%) observed for all groups.

Similar N/P ratios were used for hybrid peptides, and the effect of the addition of DOPE to the delivery system was also investigated. Both peptides demonstrated significant efficiency in delivering siRNA to different breast cancer cell lines, and similar to our observation for [WR]_5_, the addition of DOPE increased the mean fluorescence for both peptides (although not always statistically significant in MDA-MB-468 cells; Figure 3). However, the efficacy of siRNA uptake with the peptides in this class seemed to depend on the cell line: while both peptides created similar mean fluorescence readings to in MDA-MB-231 cells, addition of DOPE had a more significant effect on improving the internalization level with [R_6_K]W_6_ compared to [R_5_K]W_5_ at N/P ratios of 40 and 80 (mean fluorescence of 1359 compared to 671 for N/P ratio of 40, respectively; Figure 3A). On the other hand and in AUC65 cells, [R_5_K]W_5_ created higher mean fluorescence, both with and without DOPE (mean fluorescence readings of 1942, 921, and 1392 for N/P ratios of 20, 40, and 80 with DOPE, respectively, compared to 472, 611, and 1237 for corresponding N/P ratios for [R_6_K]W_6_; Figure 3E). However, there was no significant difference between the overall mean fluorescence values observed for the two peptides in MDA-MB-468 cells (Figure 3C). Interestingly, the effect of N/P ratio on the siRNA internalization levels was also cell type dependent: while the highest mean fluorescence was observed with highest N/P ratio in MDA-MB-468 cells (for both peptides, with and without DOPE), a similar trend was not observed in the other two cell lines. In MDA-MB-231 cells, N/P ratio of 40 provided the highest N/P ratio for both peptides (not significantly different with N/P ratio of 20 in [R_5_K]W_5_ with DOPE). On the other hand, more variability was observed in AU565 cells, where the highest mean fluorescence was observed for N/P ratios of 80 and 20 for [R_5_K]W_5_ with and without DOPE, respectively, and N/P ratio of 80 for [R_6_K]W_6_ both with and without DOPE. Statistically significant differences were less frequent in terms of percentage of the cells positive for fluorescence signal, mostly due to high percentages observed for most study groups (Figure 3B,D,F). However, a trend similar to the mean fluorescence values was observed in the percentage of fluorescence-positive AU565 cells (Figure 3F).

Overall, the performance of the peptide careers was not consistent in different cell lines. This is expected and has been reported previously by many researchers [4,25,26]. In fact, many cell lines are notorious as being “hard-to-transfect”. This is perhaps due to multiple factors, including the receptor composition on the cell membrane and growth media, among others. However, a similar trend was observed for the efficiency of each designed peptide in a different cell line, which indicates the overall efficiency of the selected approach. Among cyclic peptides, [FR]_4_ and [WK]_5_ showed no efficiency in internalizing siRNA into triple-negative breast cancer cell lines (Figure 3). This was not surprising since we observed a similar trend when [FR]_4_ was evaluated along with [WR]_5_ for delivery of fluorescent-labeled lamivudine. [FR]_4_ showed significantly less molecular transporter property when compared with [WR]_5_ [18]. Furthermore, when [FR]_4_, [WK]_5_, and [WR]_5_ were compared for self-assembly properties, only [WR]_5_ generated self-assembled nanostructures. These data indicate that an optimal balance of positive charge and hydrophobicity is required to generate self-assembled structures and molecular transporter property. The presence of indole ring of tryptophan is presumed to be critical for the generation of more efficient hydrophobic interactions with the cell membrane and self-assembly. It seems that combination of arginine and tryptophan offers characteristics that are not provided when arginine is combined with phenylalanine or in combination of lysine and tryptophan.

Overall, hybrid peptides showed a higher efficiency compared to cyclic peptides, which could be explained by their specific design. The hydrophobic portion of the peptide is completely separated in [R_5_K]W_5_ and [R_6_K]W_6_, which minimizes the risk of a steric hindrance with the interionic interaction with siRNA. [R_6_K]W_6_ showed the highest mean fluorescence in MDA-MB-231 cells, which is expected due to a higher number of primary amines (six groups compared to five for [WR]_5_ and [R_5_K]W_5_). This significant difference was not observed for the percentage of siRNA-positive cells (Figure 3B). While mean fluorescence represents the total siRNA signal associated with the cell population, each cell can be recognized as siRNA-positive with internalizing a few copies of siRNA. Therefore, mean fluorescence can vary almost without limit, where the percentage is a limited scale. We frequently observe near maximum percentages for different study groups with effective delivery systems, in which case the mean fluorescence can be used to differentiate the efficiency of different carriers used. The maximum siRNA internalization in AU565 cells was observed with [R_5_K]W_5_, which was unexpected. However, the need for optimization of the delivery system based on the targeted cell line has been emphasized by many experts, and different performance of carriers in different cell lines is somewhat expected.

The second variable in uptake studies was the N/P ratio. The overall positive charge in the peptide/siRNA complexes is contributed by the primary amine groups (which were considered ‘protonable’ in the N/P calculation method). A higher N/P ratio is usually expected to create a higher interaction with the negatively-charged cell membrane. In fact, a positive overall charge (> + 5 mV) is usually recommended for effective delivery of nucleotides [24,27,28]. It is, however, hypothesized that increased N/P ratio and resultant enhanced interaction with cell membrane also leads to increased cytotoxicity [29]. This correlation between internalization efficiency and the level of cytotoxicity is well-established in polyethyleneimine (PEI) positively-charged polymers [4,24,30]. This expected trend was observed in our siRNA internalization studies using flow cytometry for most of the study group. Increasing N/P ratio from 20 to 40 increased mean fluorescence in all study groups (in some cases the increase was not statistically significant) with one exception: [R_5_K]W_5_ in AU565 cells, where N/P ratio of 20 resulted in the highest mean fluorescence. The increase in N/P ratio from 40 to 80, however, did not always increase the level of internalization indicated by mean fluorescence, which could indicate a ‘saturation’ in the uptake of complexes at the N/P ratio of 40. The percentage of the cells positive for the fluorescent signal did not show the similar trend in all cases, which again could be due to the high level of internalization observed for N/P ratio of 20, which limited the room for improvement of the number of “transfected” cells.

The secondary structures of the peptides along with the chemical structures could contribute significantly to their bioactivity. The CD spectra of α-helices have two negative peaks of similar magnitude at 222 nm and 208 nm, and a larger positive peak at 195 nm. A ratio of 222 nm/208 nm of about 0.8 suggests a single stranded α-helix while a ratio of about 1 suggests a two-stranded coil–coil of α-helix. We have already conducted and reported CD studies for peptides containing tryptophan and arginine residues [WR] (*n* = 3–5) [19] and peptides containing tryptophan and lysine residue [WK]_5_ [31]. The secondary structures of these cyclic peptides were distinct from known α and β structures as previously reported by us [19,20]. CD spectra of the aqueous solution of [WR]_n_ (*n* = 3–5, 100 µM) showed two negative bands at approximately 202–205 nm and 214–216 nm and a positive band around 229 nm, suggesting a distorted helical structure [19]. The CD spectra of [WR]_5_ exhibited a distinct minimum of ellipticity around 202–205 nm, suggesting that the peptides exist mostly in random coil states. Furthermore, the peptide showed strong negative bands around 214–216 nm and a positive band at around 190, which are characteristic of the formation of distorted β-sheet structures [19]. CD spectra of [WK]_5_ has also been previously reported in the same publication. CD revealed a negative band at approximately 216 nm and a positive band around 230 nm. The negative 205 peak is less obvious than [WR]_5_. These CD spectra are distinct from a helical structure. In general, [WR]_5_ shows a distorted helical structure that may explain more efficient cellular permeability. This distorted helical structure is less obvious in [WK]_5_. However, neither of the two peptides demonstrated fully helical structure. We have conducted preliminary CD studies on several new hybrid peptides along with [R_5_K]W_5_ and [R_6_K]W_6_. For example, CD study on [R_5_K]W_7_ showed a negative peak at about between 222 to 227 nm that is significantly different than [WR]_5_. The peak at 222 nm is consistent with an α-helical protein, but no peak at 208 nm was observed to confirm the helical structure. It also showed positive band near 287 and 295 nm in near UV, suggesting the stacking or self-assembly of tryptophan residues. We also studied the effect of increasing the overall hydrophobicity of the delivery system by adding DOPE to the formulation. We have previously reported the enhanced cellular internalization of siRNA with increased hydrophobicity through conjugation of fatty acids to PEI 2.0 kilodaltons [24] and small positively-charged peptides [23]. It is hypothesized that increased hydrophobicity of the siRNA/carrier complex would enhance interaction with the cell membrane, which in turn is expected to improve cellular uptake of siRNA. This approach has also been reported by other researchers via conjugation of hydrophobic moieties to the carrier or the siRNA itself [32,33,34]. We have previously reported the simple addition of DOPE to the siRNA/peptide mixture as an efficient approach in enhancing siRNA internalization by human breast cancer cells [23]. In this study, the addition of DOPE created a similar enhancing effect on siRNA internalization in all study groups in a different selected cell line, which of course was not always statistically significant.

**Figure 4:**
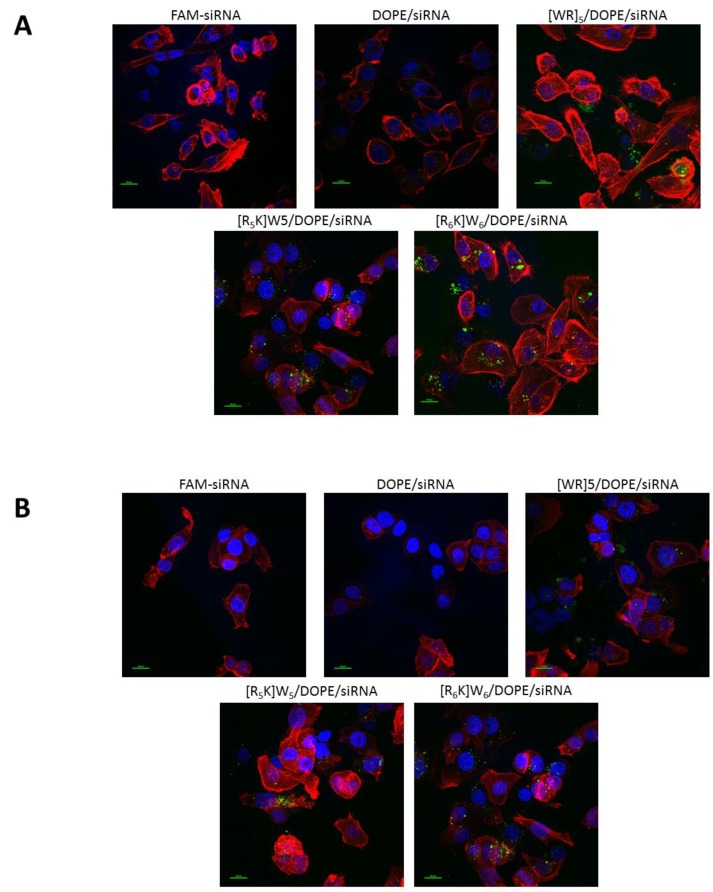
Confocal images of MDA-MB-231 (**A**) and MDA-MB-468 (**B**) cells exposed to FAM-labeled siRNA. The red, blue and green channels visualize Texas Red (used to stain the cell membrane), DAPI (stained the nuclei), and FAM-labeled siRNA.

The cell internalization of the fluorescent-labeled siRNA was also visualized using confocal microscopy (Figure 4). The margins of cytoplasm are visualized with color red (due to Texas Red staining of cell membrane), and the nuclei are visualized with a blue color (due to DAPI staining). While DOPE alone did not internalize FAM-labeled siRNA, all three studied peptides created significant internalization when combined with DOPE. In addition to visual detection of siRNA in breast cancer cells, this set of experiments confirmed that DOPE alone does not create a significant siRNA internalization, which was observed in the flow cytometry experiments, and has been previously reported for a different peptide design by our research group [23].

### 3.3. Binding Affinity

**Figure 5:**
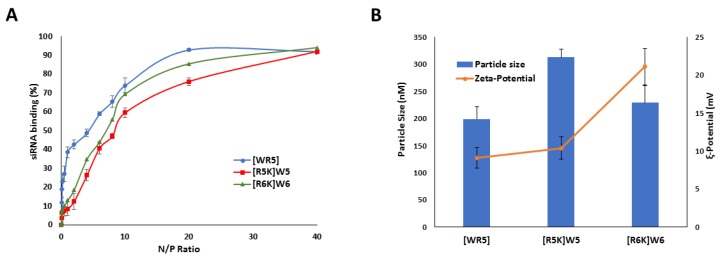
Characterization of peptide interactions with siRNA: (**A**) The binding affinity was determined at different N/P ratios to determine the N/P ratio required for 50% binding (BR50), using SYBR Green II exclusion assay (*n* = 3). Among the selected peptides, [WR]_5_ showed the highest binding affinity to siRNA; (**B**) Particle size, and surface charge of the particles were determined at an N/P ratio of 20 (Zeta Sizer; *n* = 3). [R_5_K]W_5_ and [R_6_K]W_6_ showed the highest particle size and surface charge, respectively, among the peptides included in this study.

The affinity to bind to siRNA was determined for [WR]_5_, [R_5_K]W_5_, and [R_6_K]W_6_ in a wide range of N/P ratios (Figure 5A). Complete binding was achieved with N/P ratio of 40 for all three peptides. However, the binding affinity was the strongest for [WR]_5_ (BR50 = 4.0). This could be explained by steric hindrance caused by the hydrophobic side chain on [R_5_K]W_5_ and [R_6_K]W_6_ peptides. [R_6_K]W_6_ showed a higher affinity than [R_5_K]W_5_ (BR50 of 6.9 compared to 8.3, respectively), which was also expected due to presence of six positively charged nitrogens in the structure of [R_6_K]W_6_ (compared to five positive charges for [R_5_K]W_5_).

### 3.4. Particle Size and Surface Charge

The hydrodynamic diameter and the ξ-potential of the peptide/siRNA particles were studied at N/P ratio of 20 to further characterize the peptide-siRNA interaction (Figure 5B). While the size of particles was similar for [WR]_5_ and [R_6_K]W_6_ (~200 nm), particles formed with [R_5_K]W_5_ had a larger diameter (~310 nm). However, while all particles had a positive surface charge, the ξ-potential of particles formed with [R_6_K]W_6_ was significantly higher than the other peptides. This is probably due to the extra positive charge on the structure of this peptide.

### 3.5. Cytotoxicity

**Figure 6:**
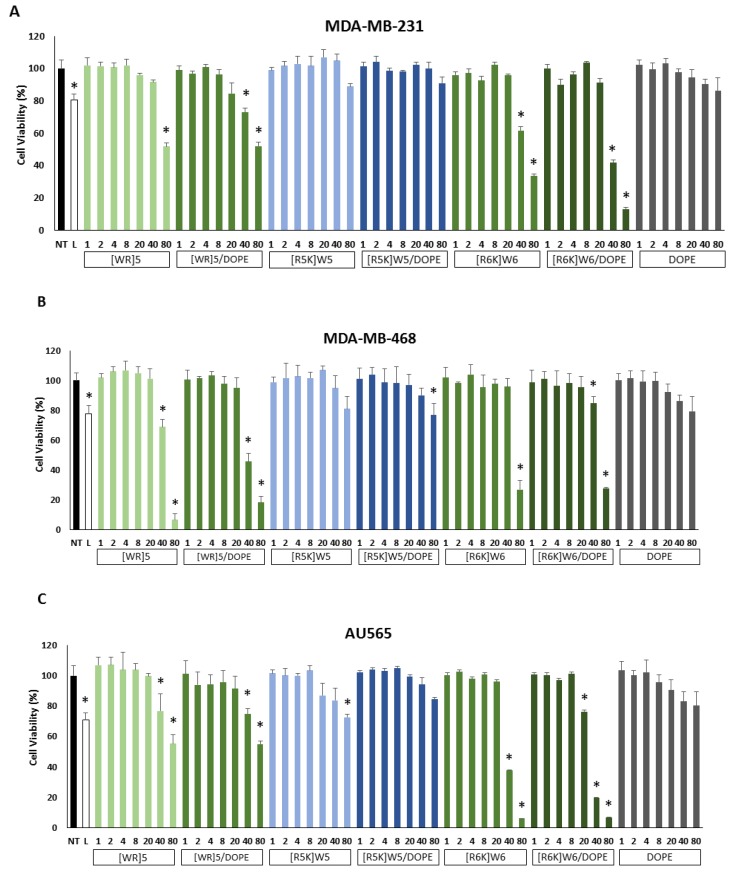
The toxicity of [WR]_5_ (with and without addition of DOPE) in (**A**) MDA-MB-231 Cells, (**B**) MDA-MB-468, and (**C**) AU565 cells with different N/P ratios (from 1 to 80) (CCK assay; *n* = 3). The viability is normalized to cells treated with normal saline (no treatment, or ‘NT’ group). DOPE study group received DOPE alone, with the same final concentration incorporated in the delivery system at corresponding N/P ratios. The error bars indicate standard deviation. Asterisks indicate significant difference (*p* < 0.05) with the ‘NT’ group.

Safety of a delivery system is a key characteristic for the clinical applications of the carrier. We investigated the toxicity of the designed peptides after complex formation with siRNA, at different N/P ratios ranging from 1 to 80, in all breast cancer cell lines included in the study (Figure 6). In MDA-MB-231 cells, [R_5_K]W_5_ showed no significant effect on the viability of the cells in the N/P range studied. However, both [WR]_5_ and [R_6_K]W_6_ showed signs of toxicity in N/P ratios of 40 (except [WR]_5_ without DOPE) and 80. The drop in viability was most significant for cells exposed to scrambled siRNA/DOPE/peptide complexes at N/P ratio of 80 (13% compared to NT group; Figure 6A). A similar pattern was observed in MDA-MB-468 and AU565 cells, where [R_5_K]W_5_ was the least toxic of the three peptides (the significant difference was observed only for N/P ratio of 80, and the lowest viability observed was 72.6% for [R_5_K]W_5_ without DOPE in AU565 cells). The level of toxicity of [WR]_5_ was most significant in MDA-MB-468 cells, where N/P ratio of 80 caused 18.2 and 6.6% viability with and without DOPE (Figure 6B). In AU565 cells, similar to MDA-MB-231, the highest drop in viability was observed for [R_6_K]W_6_ with N/P ratio of 80 (Figure 6C). Overall, the addition of DOPE did not seem to change the cytotoxicity pattern significantly for any of the peptides. Also, exposing cells to different concentrations of DOPE alone (corresponding to the final concentrations incorporated into carriers at the different N/P ratios) did not reduce cell viability in any of the selected cell lines. However, the level of effect on the viability was slightly more in MDA-MB-468 and AU565 cells for the highest concentration used. Lipofectamine ^®^ 2000 reduced the cell viability percentage in all three cell lines significantly (~75–80% compared to NT group).

To evaluate the cytotoxicity of [WR]_5_, [R_5_K]W_5_, and [R_6_K]W_6_ alone (without siRNA complexation) in healthy cells, cell viability assay was conducted in LLCPK (normal kidney cells) at the experimental concentration of 25 μM. None of the compounds showed any significant cytotoxicity at this concentration in the selected cell line (data not shown).

As emphasized before, a correlation between the efficacy of cellular internalization and cytotoxicity is usually expected for positively charged siRNA carriers. An increased cytotoxicity was in fact detected with increasing N/P ratio for all selected peptides in all three cell lines, and was most significant with N/P ratio of 80. [R_6_K]W_6_ showed the highest level of toxicity in all cell lines, which is consistent with the expectation due to the presence of six primary amine groups compared to five groups for the other two peptides. [R_5_K]W_5_ showed the least level of toxicity in all three cell lines, and in fact the cell viability was not significantly different at N/P ratio of 80 compared to the ‘No Treatment’ group in MDA-MB-231 cells. This indicates that [R_5_K]W_5_ is a more promising carrier compared to [WR]_5_ in both efficiency and safety aspects. No significant toxicity was detected at N/P ratio of 20 for any of the peptides, and therefore this ratio was selected for protein silencing experiments to avoid the interference of toxicity with the silencing effect (despite higher internalization observed for N/P ratios of 40 and 80 in most of the study groups).

### 3.6. Protein Silencing

**Figure 7:**
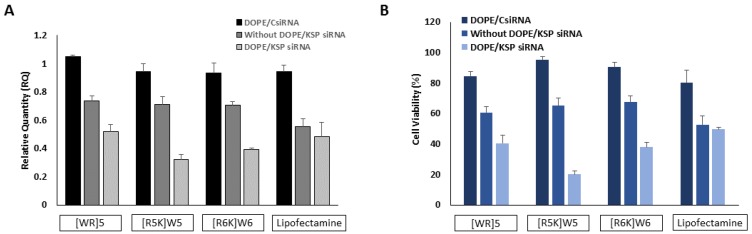
Silencing kinesin spindle protein (KSP) in MDA-MB-231 cells: (**A**) Silencing was evaluated at mRNA level using real-time PCR. Control siRNA (CsiRNA, only delivered with DOPE) or siRNA targeting selected protein (delivered with or without DOPE) were delivered using the designed peptides at N/P ratio of 20. All relative quantities were normalized based on mRNA levels of cells treated with normal saline (not shown). All relative quantities for targeted siRNAs were significantly different with CsiRNA group, and all mRNA levels for targeted siRNA groups were significantly different with CsiRNA group, and all mRNA levels for targeted siRNA groups were significantly different from corresponding ‘without DOPE’ levels (*p* < 0.05). (**B**) Effect of silencing on MDA-MB-231 cell viability was evaluated using CCK assay. Similar study groups were used, and results were normalized based on viability of the cells treated with normal saline (not shown). A similar trend in statistical analysis was observed.

We targeted two model proteins as a proof of concept using the designed peptides that showed significant uptake in human breast cancer cells. The effect of targeting KSP was analyzed at mRNA level by real-time PCR and on cell viability using CCK assay (Figure 7). Peptide/siRNA complexes were formed at N/P ratio of 20 to avoid the toxic effect on the MDA-MB-231 cells. Also, scrambled siRNA (control siRNA, or ‘CsiRNA’) was only delivered with DOPE included in the delivery system as a negative control. Delivering siRNA targeting KSP decreased the mRNA level of the targeted protein compared to the negative control group (statistically significant; *p* < 0.05). Similar to the trend observed in the cellular internalization study, the addition of DOPE increased the silencing efficiency for all selected peptides. The level of silencing was most significant in [R_5_K]W_5_/DOPE delivery system (relative quantity of 0.32; Figure 7A). The level of silencing efficiency with addition of DOPE to both [R_5_K]W_5_ and [R_6_K]W_6_ was significantly higher than Lipofectamine (used as positive control). A CCK assay was also used to investigate the viability of the MDA-MB-231 cells after targeting KSP. A very similar trend was observed in the viability pattern of the study groups with minor differences: scrambled siRNA delivered with [WR]_5_ demonstrated a slight decrease in viability of the exposed cells (85% normalized to cells treated with normal saline) that was statistically significant. Also, unlike the real-time PCR results, where [R_6_K]W_6_/DOPE showed more significant efficacy in downregulating KSP mRNA compared to [WR]_5_/DOPE study group, no significant difference was observed in the effect of the two delivery systems on the viability of the cells targeted with KSP siRNA. Again, targeting KSP by [R_5_K]W_5_/DOPE showed the most significant effect on cell viability (20.2%; Figure 7B); however, the diminished viability was more significant than Lipofectamine with both [R_5_K]W_5_ and [R_6_K]W_6_ after addition of DOPE to the delivery system.

KSP has been identified as a viable target for siRNA silencing as a therapeutic approach in cancer therapy. Delivering siRNA via a variety of delivery systems targeting KSP in different cancer types has shown effective apoptosis induction and tumor growth retardation in vitro and in vivo [35,36,37,38,39,40]. In fact, clinical trials have indicated the efficiency of simultaneous silencing of KSP and vascular endothelial growth factor (VEGF) in colorectal [41] and endometrial cancer patients [42] with liver involvement. We selected KSP as a model protein to investigate silencing efficiency of the designed selected peptides in MDA-MB-231 cells, and to determine the effect of this silencing on the cell viability as the phenotypical end result of the siRNA delivery. While delivering KSP siRNA without DOPE had a modest effect on mRNA levels as well as the cell viability for all three peptides, the addition of DOPE enhanced the effect significantly in all study groups, which was most significant for [R_5_K]W_5_ (Figure 7). This is further confirmation of the efficient siRNA delivery via the designed peptides and timely release of siRNA from the complexes in the cytoplasm. Although [R_6_K]W_6_ showed the highest level of siRNA cellular internalization among the designed peptides in this cell line with N/P ratios of 40 and 80, the lower silencing efficiency of this peptide is not surprising due to the lower N/P ratio selected to avoid non-specific toxicity.

**Figure 8:**
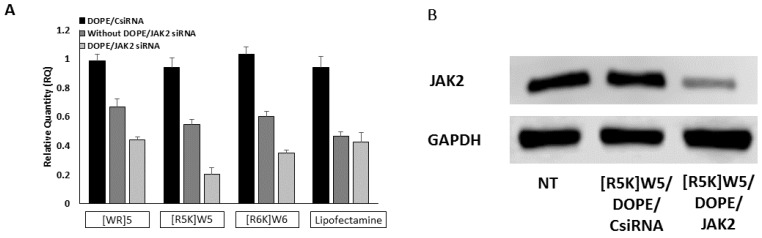
Silencing Janus kinase 2 (JAK2) in MDA-MB-231 Cells: (**A**) Control siRNA (CsiRNA, only delivered with DOPE) or targeting selected protein (delivered with or without DOPE) were delivered using the designed peptides at N/P ratio of 20. All relative quantities were normalized based on mRNA levels of cells treated with normal saline (not shown). All relative quantities for targeted siRNAs were significantly different from the CsiRNA group, and all mRNA levels for targeted siRNA groups were significantly different from the corresponding ‘without DOPE’ levels (*p* < 0.05). (**B**) JAK2 protein level was quantified for cells treated with CsiRNA and JAK2 siRNA (delivered by [R5K]W5/DOPE) using western blot.

A similar study design was used to investigate targeting JAK2 protein in the same cell line, and in addition to the real-time PCR, western blot was performed to confirm the effect of siRNA delivery on the protein expression level (Figure 8). The analysis of mRNA levels of JAK2 in different study groups revealed a similar pattern to our observation for KSP. Again, delivering siRNA without DOPE decreased the mRNA level compared to scrambled siRNA for all selected peptides, and adding DOPE to the delivery system further enhanced this effect significantly, which was again a silencing efficiency that was significantly higher than Lipofectamine, for both [R_5_K]W_5_ and [R_6_K]W_6_. [R_5_K]W_5_/DOPE again showed the most significant drop in mRNA level (~80% silencing; Figure 8A). This delivery system was selected for western blotting to confirm our observations at the protein level (Figure 8B).

In order to further confirm the silencing efficiency of peptide/DOPE/siRNA complexes, JAK2 was selected as a second model protein. JAK/STAT pathway is a crucial signaling cascade known to be involved in the proliferation and survival of different cancer cells [43]. Silencing JAK2 has reported to enhance apoptosis in human gastric cancer cells [44], and delivering JAK2 siRNA has sensitized resistant ovarian cancer cells to paclitaxel [45]. Level of mRNA downregulation showed a similar trend to our observations with KSP silencing with the most efficient drop in mRNA levels for [R_5_K]W_5_/DOPE delivery system. In order to confirm the silencing efficiency at the protein level, we performed western blotting to determine inhibition of JAK2 expression by this delivery system, which further confirmed the silencing efficiency of the selected carriers, in correlation with our observations in uptake and PCR studies.

## 4. Conclusions

It was expected that incorporation of positively-charged and hydrophobic residues in the structure could greatly improve the interaction with the negatively charged phosphate and hydrophobic chains in the cellular phospholipid bilayer, respectively, and enhance the cellular delivery of siRNA. This expectation was based on the previous data showing that cyclic peptides containing alternate tryptophan and arginine residues [WR]_5_ and [WR]_4_ and the corresponding peptide-capped gold nanoparticles improved the cellar delivery of siRNA [22]. Consistent with the previous results, we found that [WR]_5_ was an efficient transporter of siRNA. We also found that cellular delivery of siRNA was significantly improved in the presence of peptides containing arginine and tryptophan residues with a cyclic peptide containing all the positively-charged residues attached to tryptophan chain, suggesting more efficient interactions of the trypotphan chain with the hydrophobic residues and positively-charged residues of the peptide with the negatively-charged phosphate group in the phospholipid bilayer.

## Supplementary Materials

The following are available online at https://www.mdpi.com/2073-4360/11/4/703/s1.
