# Supplementary material for: Amphiphilic Peptides for Efficient siRNA Delivery
Source: Polymers (Basel). 2019 Apr 17;11(4):703. doi: 10.3390/polym11040703 (PMC6523661; doi:10.3390/polym11040703)
Supplement: Supplementary file 1 [file polymers-11-00703-s001.pdf]

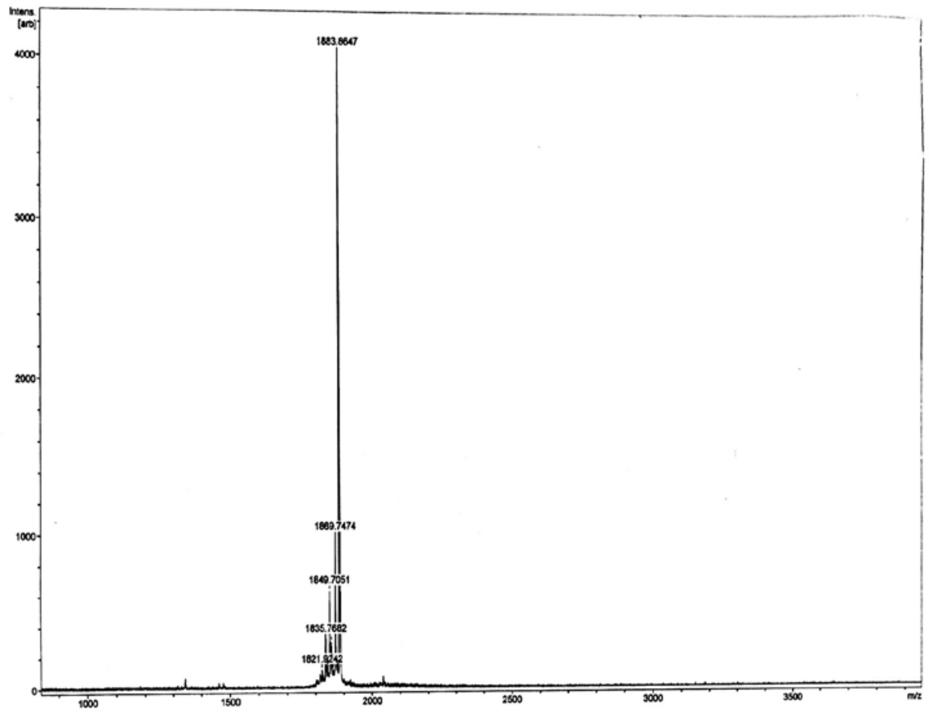

$[R_5K]W_5$: MALDI-TOF (m/z): $C_{93}H_{124}N_{32}O_{12}$, calculated: 1881.0076, found: 1883.8647 $[M+2H]^+$

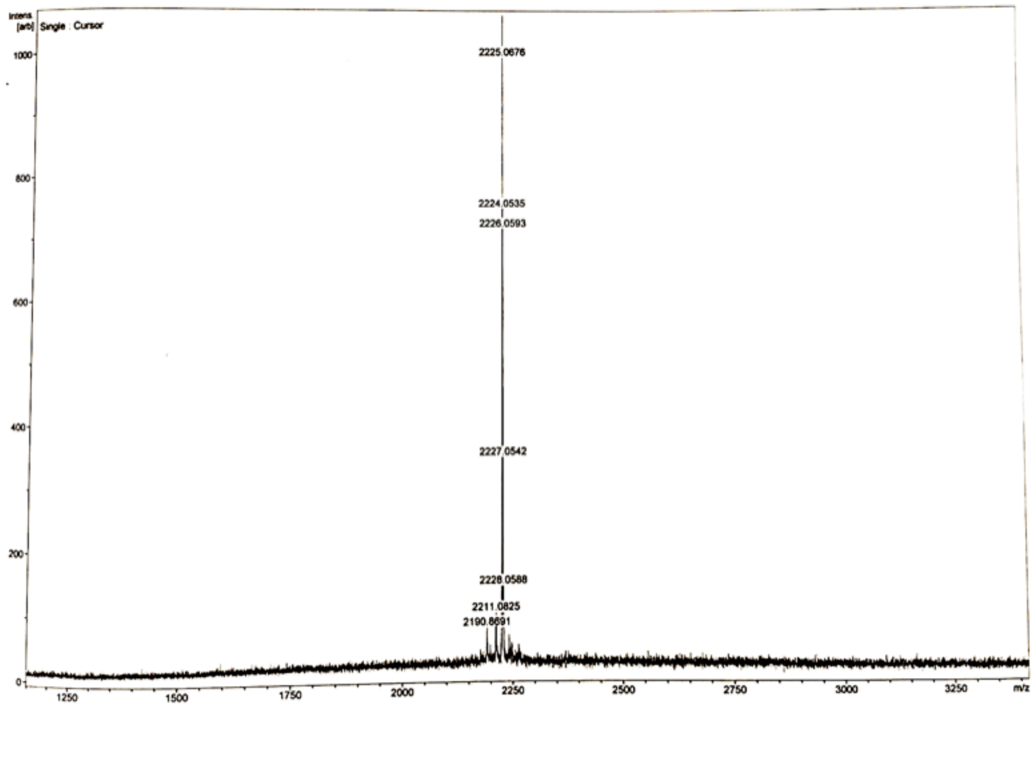

$[R_6K]W_6$: MALDI-TOF (m/z): $C_{110}H_{146}N_{38}O_{14}$, calculated: 2223.1881, found: 2225.0676 $[M+2H]^+$
